# Antibiotic Resistome Biomarkers associated to the Pelagic Sediments of the Gulfs of Kathiawar Peninsula and Arabian Sea

**DOI:** 10.1038/s41598-019-53832-9

**Published:** 2019-11-21

**Authors:** Chandrashekar Mootapally, Neelam M. Nathani, Paresh Poriya, Imtiyaz Beleem, Jignesh C. Dabhi, Indra R. Gadhvi, Chaitanya G. Joshi

**Affiliations:** 1 Department of Marine Science, Maharaja Krishnakumarsinhji Bhavnagar University, Bhavnagar, 364-001 Gujarat India; 2 Department of Life Sciences, Maharaja Krishnakumarsinhji Bhavnagar University, Bhavnagar, 364-001 Gujarat India; 3 Government Science College, Bhakta Kavi Narsinh Mehta University, Veraval, 362-265 Gujarat India; 4 Department of Animal Biotechnology, College of Veterinary & Animal Husbandry, Anand Agricultural University, Anand, 388-001 Gujarat India; 5 Gujarat Biotechnology Research Centre (GBRC), Department of Science & Technology, Government of Gujarat, Gandhinagar, 382-011 Gujarat India

**Keywords:** Marine biology, High-throughput screening

## Abstract

Antibiotic resistance has been one of the most persistent global issue. Specifically, marine microbiomes have served as complex reservoirs of antibiotic resistant genes. Molecular advancements have enabled exploration of the uncultured microbial portion from hitherto difficult to sample niches such as deeper oceans. The Gulfs of Kathiawar Peninsula have been known for their unique properties like extreme tidal variations, different sediment textures and physicochemical variations. Pelagic sediment cores across four coordinates each of the Gulf of Kutch, Gulf of Khambhat and an open Arabian Sea were collected, processed for metagenomic sequencing and assessed for antibiotic and metal resistome. The dominant genes were mostly resistant to macrolides, glycopeptides and tetracycline drugs. Studied samples divided into three clusters based on their resistome with *car*A, *mac*B, *bcr*A, *tae*A, *srm*B, *tet*A, *ole*C and *sav*1866 among the abundant genes. Samples from creek of Gulf of Kutch and mouth of Gulf of Khambhat were most diverse in resistance gene profile. Biomarkers observed include *gyr*A mutation conferring resistance gene in the Arabian Sea; *Proteobacteria* species in Gulf of Kutch and Arabian sea; while *Aquificae*, *Acidobacteria* and *Firmicutes* species in the Gulf of Khambhat. Region-wise differentially abundant 23 genes and 3 taxonomic biomarkers were proposed for antibiotic resistance monitoring.

## Introduction

Deep marine environments, though, hypothesized to be pristine niche, have been continuously reported to host a reservoir of antibiotic resistant microbial community and their corresponding antibiotic resistant genes (ARGs)^1^. The water bodies receive the antibiotic contaminants from the surrounding niche thus making it a reservoir to newly resistant microbes^2^. Studies have observed that the geographical location influence the resistome patterns in the sludge samples and the microbiota composition shifts were further influenced^3^. However, few of the dominant genes in the treatment plant samples, sludge like environments and sediments were common such as those against the tetracycline class of antibiotics. Thus, a snapshot on the resistance gene pattern will provide detail on the status of the water bodies and requirement of the control strategies for preserving the water resources. Current research challenges mainly include the need to achieve deep insights into the structural and functional dynamics of microbiota by proper inference of the experimental and computational metagenomic data^4^ by ensuring the reproducibility of the conclusions drawn from metagenomic studies of complex environments. Linear discriminant analysis (LDA) effect size (LEfSe), that provides biological explanations for establishing the link between statistical significance to the biological stability and allows effect size estimation of predicted biomarkers, has been often applied to detect differentially abundant microbial features related to specific metabolic roles in specific niches^5^. This in turn provides an explanation of the biological reasons behind the persistent differences in microbial communities and their abundances. Initial reports on the potential of microbial communities to be used as biomarkers for niche/host specific factors was proposed more than a decade ago^6^.

With the advancement of molecular omics, biomarker discovery is currently one of the most preferred way to interpret the -omics data into field applications^5,7^. Biomarkers have been widely used as a signal of environmental contamination in risk assessment and strategy developments for environmental protection^8^.Until recently, metagenomic studies involving ARGs have majorly focused on the holistic analysis of ARGs based on the antibiotic classification and very few have provided the inter-relationship between the ARGs and their encoding microbial community. Though huge number of expeditions are undertaking high throughput studies of various oceanic locations, to the best of our knowledge, the pelagic microbial community from the Indian waters have not been yet explored. Specifically, meagre studies are yet performed that have reported the occurrence and distribution of ARGs and corresponding microbial community in the Indian Gulfs, especially the pelagic sediments of the Gulf of Khambhat and Gulf of Kutch. We recently reported the ARG distribution in the Gulf of Khambhat and Arabian Sea^9^, and hereby extend the work by providing the antibiotic resistance biomarkers from the pelagic sediments of the Gulf of Khambhat and Arabian Sea along with the Gulf of Kutch.

Gulf of Kutch which is semi-enclosed and an inverse estuary, has been often explored for its dynamics, tidal circulations and thermohaline structures^10,11^ due to its highly energetic macro-tidal system. It has been reported that the Indus river is the only river that indirectly contributes to the Gulf sediments by way of long currents from the mouth region^12^. Several industrial plants including fertilizer, chemicals, oil refineries, power plants, salt workers use the water, however despite this, the waters are reported to be healthy and hosting a rich biodiversity^12^. Thus, the Gulf of Kutch (GOK) possesses quite distinct niche features when compared to the Gulf of Khambhat (GOC), as well as has slightly varied on shore activities when compared to the GOC and the Arabian sea (A) coastline of the Kathiawar peninsula. The deep sediments are predicted to be pristine environment; however, recent molecular studies have revealed that even the deeper sediments host a huge amount of novel antibiotics and a good number of resistance genes^1,9^. In lieu of the same, we hypothesized the presence of enriched resistome in the pelagic sediments of the Gulfs in the Kathiwar peninsula, which harbour unique sedimentation process as detailed formerly and varied anthropogenic activities are being carried out on their shores. These activities may also play role in the variation across the Gulf’s resistome and subsequent microbiota. The pelagic sediment cores were collected from 4 coordinates across the GOK and studied the antibiotic as well as metal resistome profile of the same. We also compared the ARGs and corresponding microbial community between GOK, GOC and A metagenomic samples. Further, we also performed LEfSe analysis to discover the specific genes as well as microbial species (biomarkers)which can explain the different ARG profile and abundance in the respective niche characteristic. The study explored the ARGs distribution across the Gulfs and Arabian sea to assess the influence of niche specific characters by means of bacteria and ARG biomarkers.

## Results

### Physicochemical properties of the sediment samples

The sediment samples were assessed for their properties such as pH, salinity, Total Dissolved Salts (TDS), Electric Conductivity (EC), Resistance and Total Organic Carbon (TOC) (Supplementary Table 1). We could observe that the pH was alkaline in all the sediment samples with on an average 8.3 to 8.9 in each site (Supplementary Fig. 1). The TOC varied with highest value in the sample A compared to sediments from both the Gulfs and the salinity was almost similar in the A, TMS samples of GOK (S1 & S2) and GOC (S3 & S4) samples, while the AFM samples in GOK (S3 & S4) had significantly high salinity. AFM samples in GOC had varied values with slightly higher salinity in GOCS1 sample compared to GOC-TMS and relatively lower in the GOCS2 sample.

### Metagenomics data analysis

The sediment samples were shotgun sequenced and 160 to 222 million of paired-end reads were obtained with ~97% raw reads having a Q-score more than 20. Each sample was De Novo Assembled and the statistics of the assembled sequences are as described in Table 1. The statistics for the A and GOCS1-4 were not shown here to avoid redundancy of the results. The reads for the GOKS1-4 were submitted to EBI metagenomics under the project accession number PRJEB26615.

**Table 1:** Assembly statistics of shotgun metagenomic samples.

Sample	Input reads	Assembled Bases (Mb)	N50	No. of contigs	Largest contig size (Kb)
GOKS1	193,160,306	170	2184	79465	194
GOKS2	160,228,208	208	3226	77371	219
GOKS3	201,211,622	397	3116	148243	298
GOKS4	222,835,532	353	2686	143556	361

#### Sediment antibiotic and metal resistome

The resistome analysis revealed that considering all the nine samples, we observed hits against a 2367 ARGs that included the predicted genes encoded by different organisms for antibiotic resistance as well as the validated mutations in genes that conferred resistance to the microbe. Among them, hits to 2355 ARGs were encoded by the sediment microbiota in the four samples from GOK; 2353 by the GOC microbiota and the A had hits against a set of 2292 ARGs. The ARGs had a length between 18 to 400 amino acid with an average length of 200 amino acid in the studied samples. On the whole, it was observed that GOK was most diverse and the Gulfs were relatively more diverse than the A sample. In GOK, AFM samples were found to be more diverse with highest in the ARGs observed in the GOKS4 sample followed by GOKS3 > GOKS1 > GOKS2. Among all the studied samples, the diversity observed was GOKS4 > GOKS3 > GOCS4 > A > GOCS3 > GOCS2 > GOKS1 > GOKS2.

**Figure 1:**
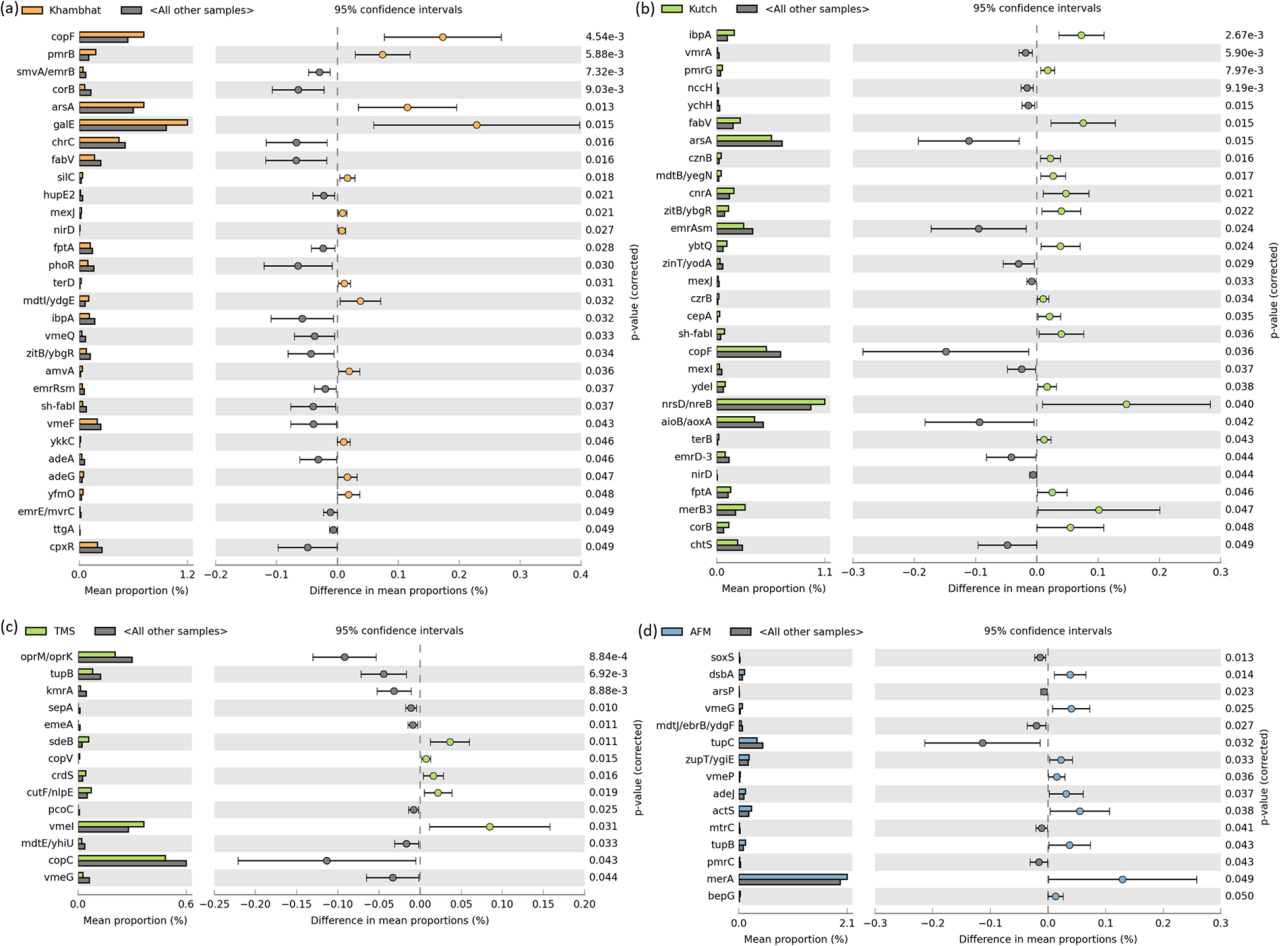
Bacteriocide and metal resistant gene difference as computed by STAMP using the Welch’s t-test representing a minimum variation at a significant level [p (corrected) <0.05] between (**a**) Khambhat and other regions, (**b)** Kutch and other regions, (**c)** TMS and other samples (**d**) AFM and other samples.

**Figure 2:**
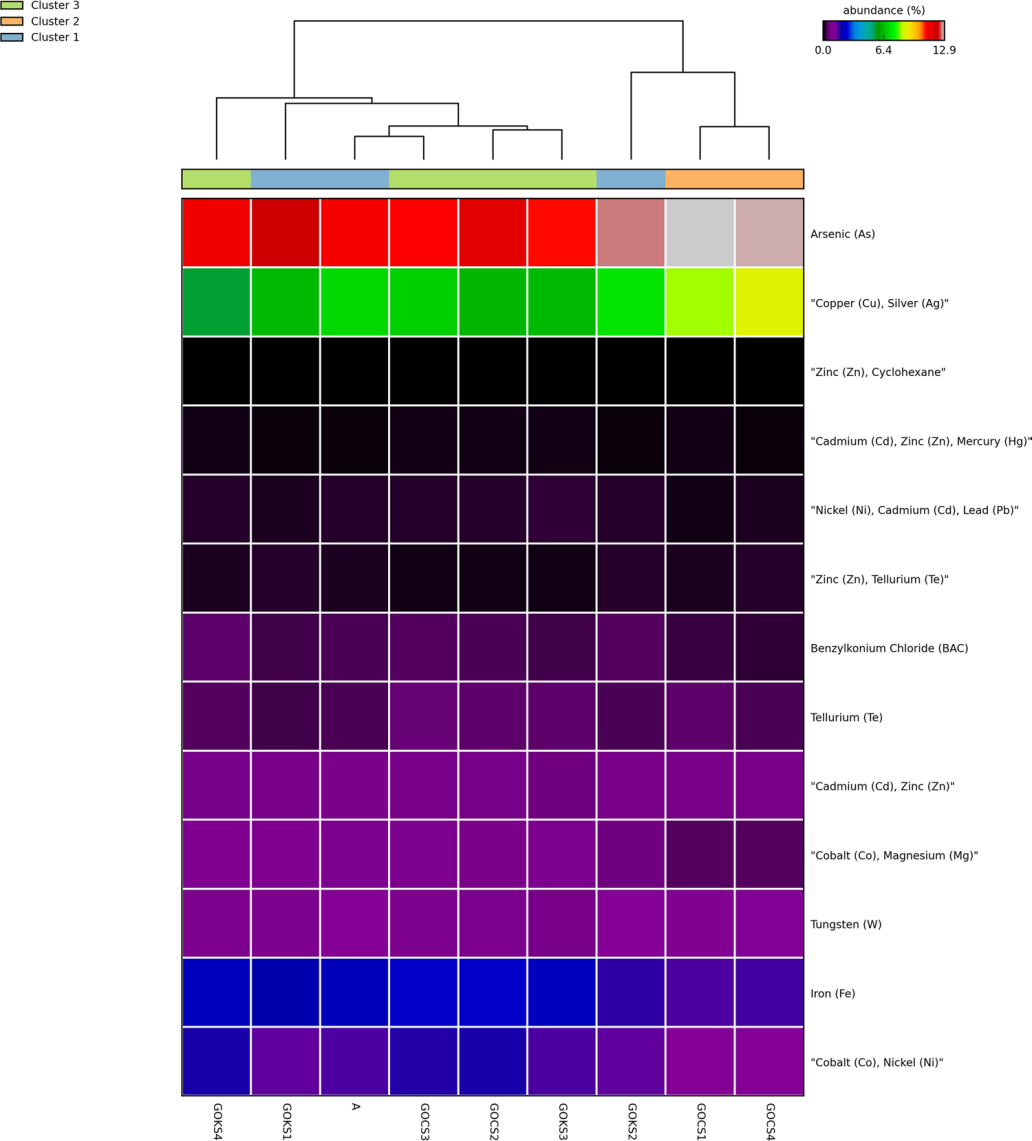
Thermal dendogram of metal resistant genes with samples grouped by similarity (UMPGA clustering) and significant genes (p < 0.05 between the three clusters as obtained based on drug class) ranked by their mean abundance.

The maximum hits against the bacteriocidal and metal resistance genes was observed in the GOKS3 sample with near similar proportions in all sample except GOKS1 and GOKS2 which had relatively lesser proportion of hits (~25% lesser hits) (Supplementary Fig. 2). In contrast to ARGs, diversity of metal resistant genes was more in the GOC and A compared to most of samples of GOK with GOKS3 as an exception. When looking into the three sites, Khambhat had few genes corresponding to resistant to Copper, Arsenic and Cetyl trimethyl ammonium bromide (CTAB) significantly varying from other two sites with slightly higher proportion of the same, while genes against multiple metals were lesser in proportion (Fig. 1a). In the Kutch samples, genes against the Cobalt and Nickel were significantly higher compared to other sites (Fig. 1b). On the whole, Iron chelation related genes were enriched in the Gulfs with genes against organo-sulfate and acid molecules being significantly varying between the TMS compared to AFM and open ocean regions (Fig. 1c,d). On the whole, considering the cluster comparison, genes conferring resistance to Arsenic, Copper and Silver were most dominant and significantly varying between the clusters (Fig. 2 and Supplementary Fig. 3). From the total genes, ~3–6% of the genes were those with MDR like predicted efflux pump related functions and 7–10% were efflux transport mechanism based functioning genes corresponding to Acriflavine, Arsenic (As), Cetrimide (CTM), Cetyl trimethyl ammonium bromide (CTAB), Chlorhexidine, Ethidium Bromide, Hydrochloric acid (HCl), Lead (Pb), Nickel (Ni), Silver (Ag), Sodium Cholate, Sodium Deoxycholate (SDC), Triclosan, Triton X-100, Zinc (Zn) and other similar multiple compounds reflecting the effect of these compounds in the environment in co-selection of antibiotic resistance.

**Figure 3:**
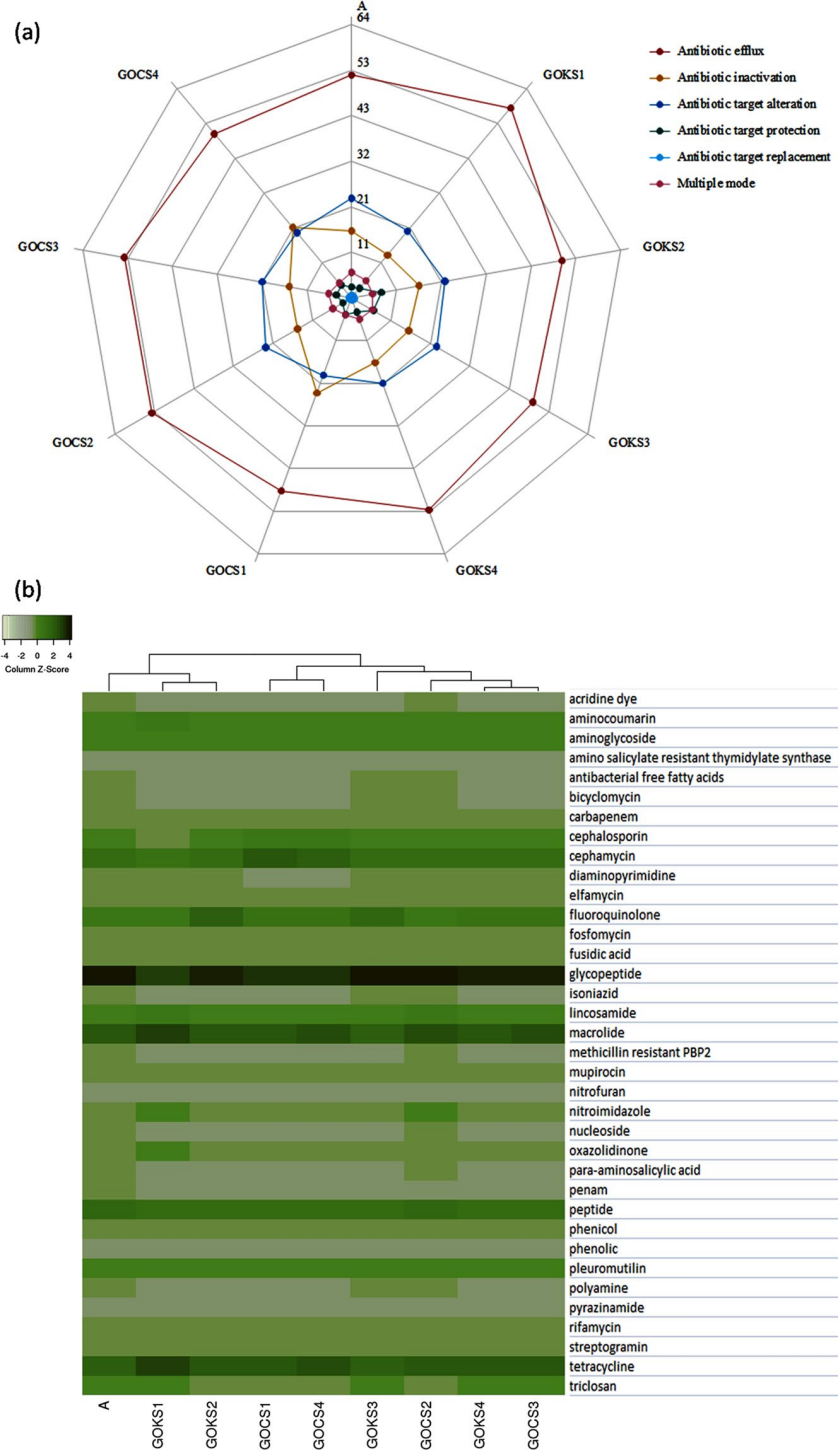
(**a**) Proportion (%) of the antibiotic resistant mechanism of observed ARGs in studied sites (**b**) Relative abundance of drug classes among the studied samples.

The mode of actions by which the microbiota confer the antibiotic resistance were similar in trend for all the studied sediment samples (Fig. 3a). The antibiotic efflux was the most dominant mode of action (>45%) in all the samples, followed by the antibiotic target alteration in the GOK and A samples; whereas the GOC samples had almost equal proportions of ARGs using the antibiotic target alteration and antibiotic inactivation in half of the samples. Almost 5% of the predicted resistance genes were classified under those that are reported to employ multiple mechanisms of action to confer resistance. ARGs conferring resistance by antibiotic target protection and target replacement based mechanism were among the lower proportions in the studied samples.

The samples formed three significant clusters when assessed for the antibiotic type that the ARGs were resistant to (Fig. 3b). The A and GOK-TMS sediment samples formed a separate cluster from the rest of the samples. Among the rest of the samples, GOCS1 and GOCS4 formed the cluster 2, while the remaining four samples formed the cluster 3. The ARGs against multiple drugs were almost >40% in all the samples (Data not shown), followed by Glycopeptide resistant ARGs with >10% of the total ARGs, with relatively higher abundance in the A, GOKS3 and GOCS2 samples. The latter samples had similar trend in many of the drug class groups. Macrolide and tetracycline were the next most abundant drug classes in all the samples. The hits corresponding to the ARGs against the remaining class of antibiotics including carbapenem, fosfomycin, fusidic acid, murpirocin, nitrofuran, phenicol, phenolic, pleuromutilin, pyrazinamide, rifamycin and streptogramin were in similar proportions across the samples with the set of genes in each class corresponding to the niche and microbiota encoding them. GOCS1 and GOCS4 had lower abundance of diaminopyrimidine, while relatively higher of cephamycin. GOKS1 and GOCS2 had similar proportions of few groups such as relatively higher ARGs against nitroimidazole and macrolides. On the whole, ARGs were distributed against 36 different drug classes.

**Figure 4:**
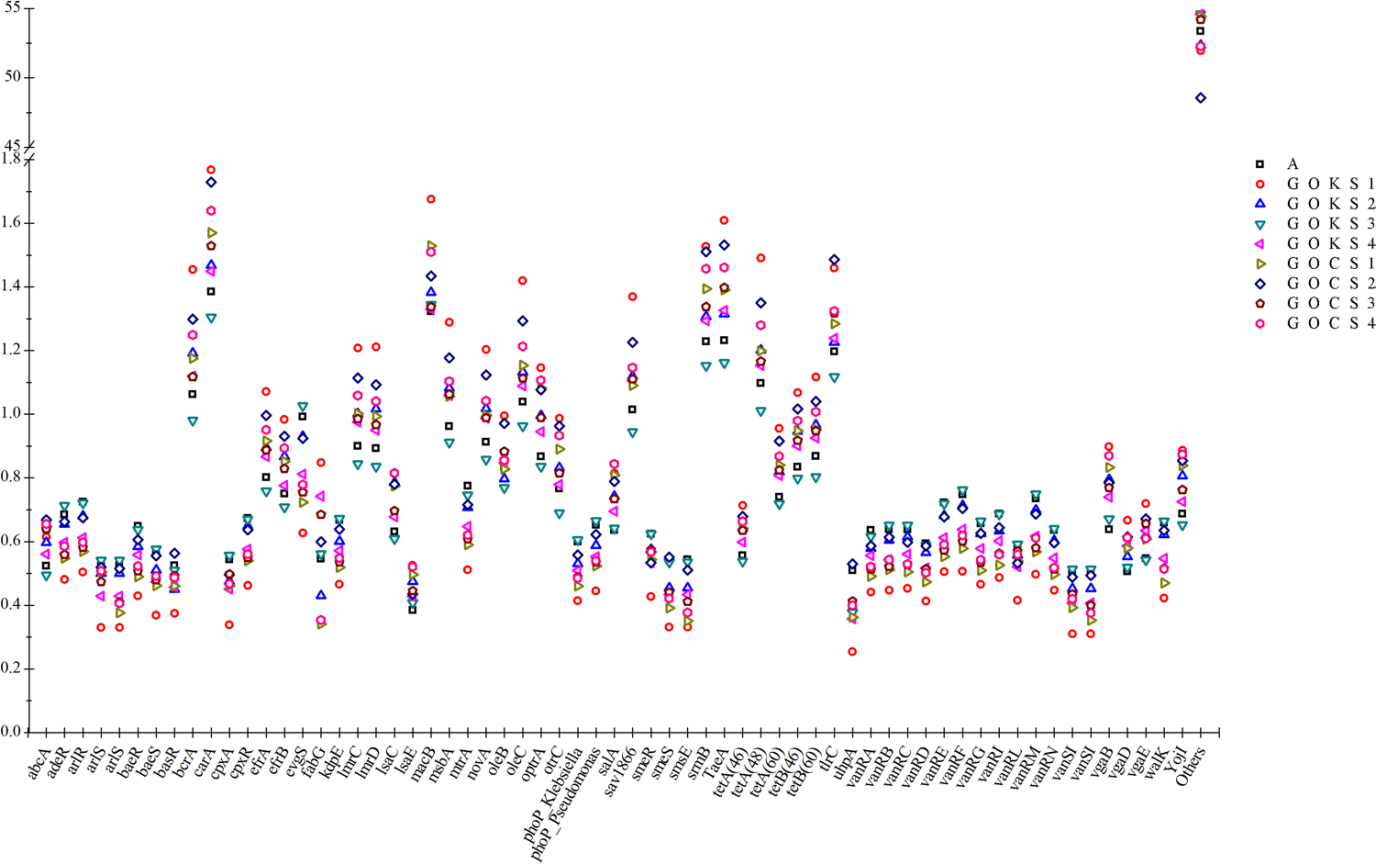
Distribution of top 63 ARGs across the sites (the depicted ARGs were those that were having at least an abundance of >0.5% in one of the nine samples) (% Abundance in Y-axis versus ARG names in X-axis).

There were 63 genes that had a percent abundance of >0.5 in at least one of the studied nine samples (Fig. 4). Among these the *car*A gene was most abundant in all the samples, followed by *mac*B. Apart from these, *bcr*A, *tae*A, *srm*B, *tet*A, *ole*C and *sav*1866 were also relatively abundant and varied in proportion between the samples. While the rest of the genes were tightly within an equal range in the samples as for example *abc*A gene proportion ranged between 0.5% to 0.7% in the studied samples. The genes apart from these 63 accounted for 49–55% (shown as others in Fig. 4) of the total ARGs abundance in each sample.

Further, when the absent ARGs from the total ARG diversity of all studied samples (2367 genes) were assessed for five sample/groups viz., A, GOK-TMS, GOK-AFM, GOC-TMS, GOC-AFM; we observed that maximum unique ARO categories were absent in the GOK-TMS samples, while no unique ARO was absent in the GOK-AFM samples (Supplementary Fig. 4). There were 30 such ARGs that were commonly absent in A and the rest of the groups. GOC-AFM group had 45 unique ARGs absent and GOC-TMS had five.

#### Marine microbial community corresponding to the resistome

**Figure 5:**
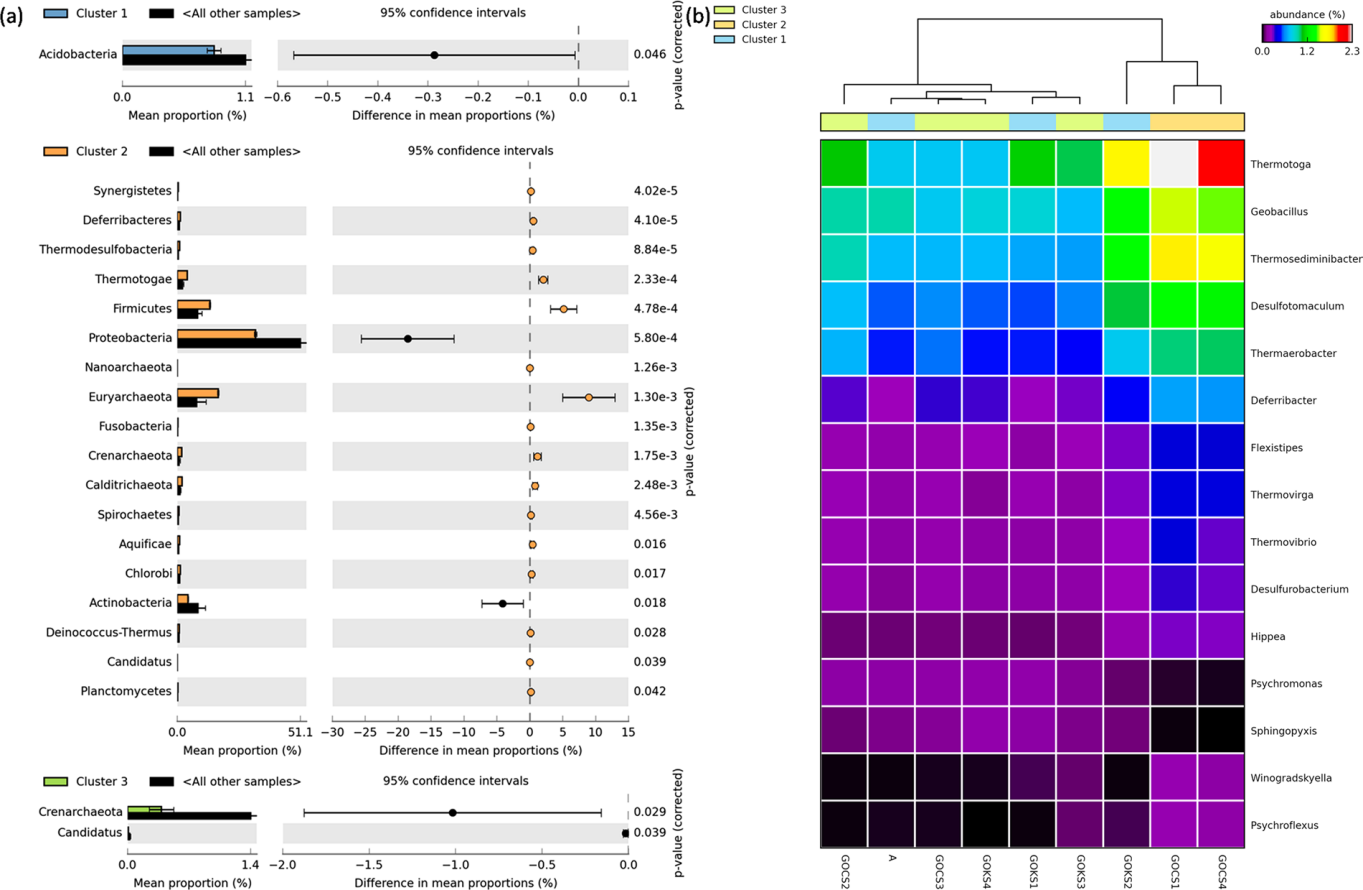
(**a**) Phylum-level difference as obtained based on drug class output as computed by STAMP using the Welch’s t-test representing a minimum variation at a significant level [p (corrected) <0.05] between: Upper - the cluster 1 against rest Middle - cluster 2 against rest, Lower -cluster 3 against rest, (**b**) Thermal dendogram of genus level abundance of microbial community with samples grouped by similarity (UMPGA clustering) and significant genera (p < 0.05 between the three clusters as obtained based on drug class) were ranked by their mean abundance.

The contigs having positive ORFs with the resistance genes were assessed for taxonomy using the MarRef database. *Proteobacteria* was the most dominant phylum in all the studied samples, followed by second most abundant phylum: *Euryarchaeota* in GOKS2, GOCS1, GOCS2; *Actinobacteria* in A, GOKS1, GOCS2, GOCS3 and *Bacteroidetes* in GOKS3 (Supplementary Table 2). While GOKS4 had *Firmicutes* as second most abundant phylum with *Actinobacteria* and *Bacteroidetes* in almost similar proportions (Supplementary Table 2). As observed in the ARG analysis, based on the resistant drug class 3 major clusters (Cluster 1: A, GOKS1, GOKS2; cluster 2: GOCS1, GOCS4; cluster 3: GOKS3, GOKS4, GOCS1, GOCS2) were formed among the nine sample (Fig. 3b). The taxonomic abundance profiles were checked for significant difference at phylum level between the clusters. The results revealed that the Cluster 2 had the highest number of significantly varied phyla (n = 18 at p < 0.05) compared to the other clusters (Fig. 5a), wherein the proportion of the commonly abundant *Proteobacteria* and the *Actinobacteria* was relatively lesser in the samples than the other clusters. *Firmicutes*, *Thermotogae* and few of the archaea phyla were slightly higher in proportion in the Cluster 2 samples. For the Cluster 1 samples, *Acidobacteria* proportion was significantly low, while the Cluster 3 had lower proportions of *Candidatus* and *Crenarchaeota* phyla (Fig. 5a).

Further, at genus level, the samples showed similar trend to the phyla level assessment, however, showed slightly different trend compared to the drug class analysis, wherein the GOKS2 (which was falling under Cluster 1 as per drug class analysis) was observed to be falling with the samples GOCS1 and GOCS4 i.e. Cluster 2 (Fig. 5b). Most of the significantly active genus (n = 15) observed in the three clusters were extremophiles, with nearly equal proportions of gram-positive and gram-negative bacteria. *Thermotoga*, *Geobacillus*, *Thermosediminibacter*, *Desultomaculum* and *Thermaerobacter* were among the higher proportions.

#### Region specific biomarkers

**Figure 6:**
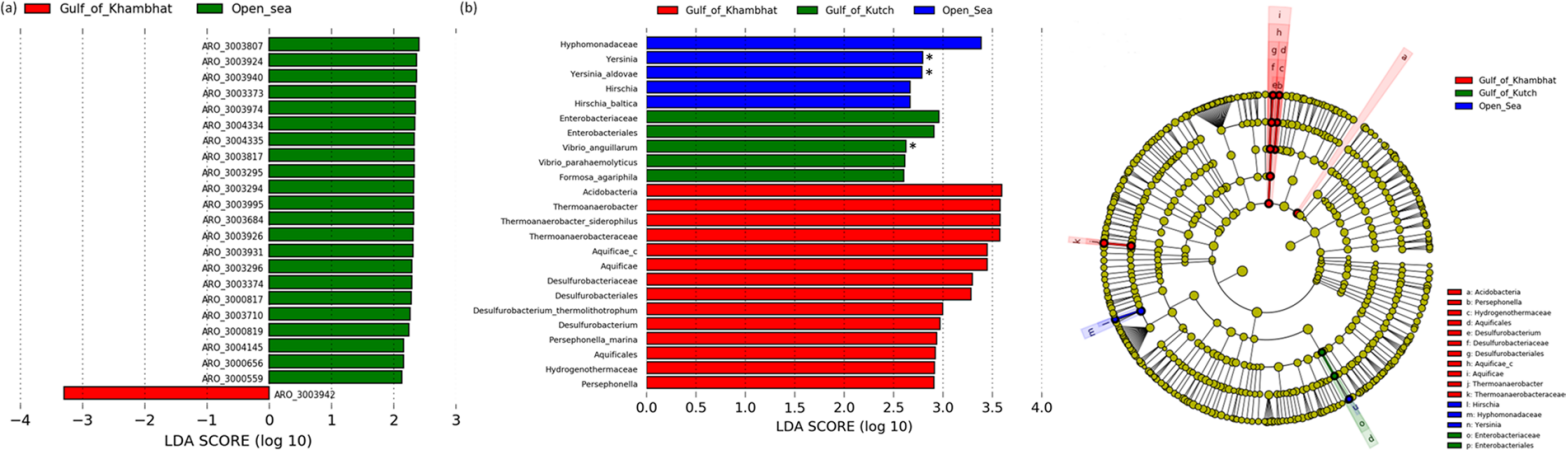
(**a**) Region specific biomarker ARGs (BM-ARG) as analyzed by LefSe as differentially abundant ARGs by the Kruskal Wallis test (p < 0.05) and LDA score >2.0, (**b**) Region specific biomarker bacterial communities (BM-BC) Left - Differentially abundant microbial taxa at different levels of classification as analyzed by LefSe using the Kruskal Wallis test (p < 0.1; p < 0.05 marked with *) and LDA score >2.0; Right - Cladogram of the differentially abundant microbial taxa, the root of the cladogram denotes domain, all the taxonomic levels depicted (up to genus) are abbreviated, different colours indicate the region with most abundance of the biomarker and the size of each node represents their relative abundance.

We assessed the presence of specific ARO as well as microbial community at different classification levels for the two Gulfs and Open sea using LEfSe. In total, 23 ARO were found to be having LDA >2.0 between the GOC and A samples (Fig. 6a), as the difference between GOC and A was more than that with either of them with GOK, thus specific marker for GOK was not observed. Only the ARO:3003942 was higher in abundance in GOC compared to both GOK and A samples, which is an ATP-binding cassette (ABC) antibiotic efflux pump, viz., *abc*A, a multidrug resistant ABC transporter that confers resistance to methicillin, daptomycin, cefotaxime, and moenomycin. All the other ARO that were less abundant in the GOC belonged to two main groups viz., the resistance-nodulation-cell division (RND) antibiotic efflux pump conferring multi-drug resistance and the *gyr*A genes resistant to fluoroquinolone, nybomycin and triclosan (Table 2). Among these, ARO:3003374, ARO:3003374 and ARO:3003940 were also significantly higher in GOK compared to GOC (Individual biomarker proportions shown in Supplementary Fig. 5).

**Table 2:** Detailed description of the Biomarker ARGs observed using LefSe.

ARO	Gene Description	Target Antibiotic/s	Mechanism
ARO:3003807	acrR	tetracycline antibiotic, phenicol antibiotic, rifamycin antibiotic, glycylcycline, cephalosporin, penam, fluoroquinolone antibiotic, triclosan	antibiotic target alteration, antibiotic efflux
ARO:3003924	*Haemophilus parainfluenzae* gyrA conferring resistance to fluoroquinolones	fluoroquinolone antibiotic, nybomycin	antibiotic target alteration
ARO:3003940	*Shigella flexneri* gyrA conferring resistance to fluoroquinolones	fluoroquinolone antibiotic, nybomycin	antibiotic target alteration
ARO:3003373	acrR	tetracycline antibiotic, penam, glycylcycline, phenicol antibiotic, rifamycin antibiotic, cephalosporin, fluoroquinolone antibiotic, triclosan	antibiotic target alteration, antibiotic efflux
ARO:3003974	*Propionibacterium acnes* gyrA conferring resistance to fluoroquinolones	fluoroquinolone antibiotic, nybomycin	antibiotic target alteration
ARO:3004334	*Salmonella enterica gyrA* with mutation conferring resistance to triclosan	triclosan	antibiotic target alteration
ARO:3004335	*Escherichia coli gyrA* with mutation conferring resistance to triclosan	triclosan	antibiotic target alteration
ARO:3003817	*Acinetobacter baumannii* gyrA conferring resistance to fluoroquinolones	fluoroquinolone antibiotic, nybomycin	antibiotic target alteration
ARO:3003295	*Mycobacterium tuberculosis* gyrA conferring resistance to fluoroquinolones	fluoroquinolone antibiotic, nybomycin	antibiotic target alteration
ARO:3003294	*Escherichia coli* gyrA conferring resistance to fluoroquinolones	fluoroquinolone antibiotic, nybomycin	antibiotic target alteration
ARO:3003995	*Clostridium difficile* gyrA conferring resistance to fluoroquinolones	fluoroquinolone antibiotic, nybomycin	antibiotic target alteration
ARO:3003684	*Pseudomonas aeruginosa* gyrA conferring resistance to fluoroquinolones	fluoroquinolone antibiotic, nybomycin	antibiotic target alteration
ARO:3003926	*Salmonella enterica* gyrA conferring resistance to fluoroquinolones	fluoroquinolone antibiotic, nybomycin	antibiotic target alteration
ARO:3003931	*Capnocytophaga gingivalis* gyrA conferring resistance to fluoroquinolones	fluoroquinolone antibiotic, nybomycin	antibiotic target alteration
ARO:3003296	*Staphylococcus aureus* gyrA conferring resistance to fluoroquinolones	fluoroquinolone, nybomycin	antibiotic target alteration
ARO:3003374	*Enterobacter aerogenes* acrR with mutation conferring multidrug antibiotic resistance	tetracycline antibiotic, penam, glycylcycline, phenicol antibiotic, rifamycin antibiotic, cephalosporin, fluoroquinolone antibiotic, triclosan	antibiotic target alteration, antibiotic efflux
ARO:3000817	mtrR	penam, macrolide antibiotic, antibacterial free fatty acids	antibiotic efflux
ARO:3003710	MexL	tetracycline antibiotic, macrolide antibiotic, triclosan	antibiotic efflux
ARO:3000819	nalD	peptide antibiotic, sulfonamide antibiotic, diaminopyrimidine antibiotic, penam, cephalosporin, phenicol antibiotic, tetracycline antibiotic, aminocoumarin antibiotic, carbapenem, fluoroquinolone antibiotic, macrolide antibiotic, monobactam, penem, cephamycin	antibiotic efflux
ARO:3004145	AxyZ	cephalosporin, macrolide antibiotic, aminoglycoside antibiotic, fluoroquinolone antibiotic, tetracycline antibiotic	antibiotic target alteration, antibiotic efflux
ARO:3000656	acrS	tetracycline antibiotic, glycylcycline, rifamycin antibiotic, phenicol antibiotic, fluoroquinolone antibiotic, penam, cephamycin, cephalosporin, triclosan	antibiotic efflux
ARO:3000559	adeN	carbapenem, penem, tetracycline antibiotic, phenicol antibiotic, diaminopyrimidine antibiotic, rifamycin antibiotic, lincosamide antibiotic, fluoroquinolone antibiotic, cephalosporin, macrolide antibiotic	antibiotic efflux
ARO:3003942	abcA	peptide antibiotic, cephalosporin, penam	antibiotic efflux

Total 24 microbial taxa were having an LDA >2.0 among the three regions under study (Fig. 6b). However, there were 3 significant markers (p < 0.05) falling under the *Proteobacteria* phyla. Among others with lower significant impact were biomarkers from the genus *Hirchia* falling under the *Proteobacteria* phyla; one species from the *Bacteroidetes* and remaining 14 biomarkers in the GOC were from three phyla viz., *Acidobacteria, Aquificae* and *Firmicutes*, and this region was exception with no biomarker reported from the *Proteobacteria* phylum.

## Discussion

Since past few years, researchers have been witnessing that microbiota not only attain antibiotic resistance on exposure to antibiotics that are a part of normal households and health centers, but they also gain their antibiotic resistome from natural environment via horizontal gene transfers or in response to different kinds of stress imposed on them by their niche^7^. Recently, with the advancement of molecular techniques for microbiome exploration, studies have been assessing relation between metals and antibiotics which has revealed that antibiotic resistance in bacteria involves co-resistance to metals^13^. In context to this, we performed resistome analysis of pelagic sediment microbiota across four coordinates in the Gulf of Kutch and compared it with similar observations in the other Gulf of Kathiawar peninsula i.e. the Gulf of Khambhat and a sample from the open sea (Arabian sea). We extended the interpretations to propose microbial and functional biomarkers specific to the three regions.

As the two Gulfs and the coastline of Arabian sea from the Kathiawar peninsula are known for their unique attributes in terms of sedimentation, salinity and on-shore activities, the physicochemical parameters of the samples were assessed. The outcomes revealed a specific trend for percentage salinity which was relatively higher in the AFM samples of the GOK (Supplementary Fig. 1). Similar proportions have been observed and reported earlier and the higher salinity could be attributed due to its inverse estuary property^11^. Also, owing to its characteristic for harboring rich biodiversity, the %TOC of the GOK sample was slightly more than GOC and the sample near to the Gulf mouth i.e. GOKS1 had similar value to the open sea sample. These parameters seem to be the reason behind several of the results observed further in the study.

The present study, to the best of our knowledge, provides a preliminary metagenomics outlook in the deeper marine sediments of the sampled region, hence the main objective was to observe the proportion of coding genes in this metagenomes against reported antibiotics and metals. Therefore, though recent studies have used a minimum cut-off of 80%, in our study, the genes with percent identity 50 as minimum were considered so that any signatures of resistant genes are not missed^14,15^. Previously, it was reported that at lower identity cut-off the sensitivity for true positives reduced, but still there was at least half the proportion was truly predicted^14^. It can be hypothesized to be more sensitive in case of marine niche which is well known for novel and rapid changes in coding genes and highly prone to horizontal gene transfers. Further, PCR based validation and confirmation would enhance the knowledge of the newly acquired mutations in the studied metagenomes and their role in the resistance. Among the mechanisms employed by the ARGs against the antibiotic target molecules, the antibiotic efflux was almost used by 48–54% of the genes in all the nine studied samples (Fig. 3a)^9^. The antibiotic efflux has been observed to be the dominant mode used in earlier resistome studies from similar niche, as the mode allows removal of target molecules from the intracellular compartments of the microbes^2,16^. The efflux pump related genes may be involved in co or cross-resistance with metal resistance. However, the second most abundant mechanism used was different from the former mentioned study, wherein they observed antibiotic target replacement as dominant which was quite less in percentage in our samples. Possible reasons for this varied observation could include different sequencing platforms used, difference in the database or database versions, difference in the sites of study, etc. The results were somewhat in line with those observed in the Antartica samples, wherein they reported varied antibiotic inactivation modes^17^, however, we also observed antibiotic target alteration as the second most abundant mode except for the GOCS1 and GOCS4 samples.

Unlike the GOC samples^9^ and similar to previous reports^18^, there was a trend observed in the GOK samples as we moved from AFM to the TMS samples, in terms of both the drug classes and the ARGs (Fig. 3b). The trend observed could be owing to the relatively less discharge rate by rivers in the GOK compared to the GOC, leading to a more stable core microbiome from creek to mouth side based on its niche parameters and nearby on-shore activities. Also, similar to most of sediment studies, the multi-drug resistant (MDR) genes were most abundant^9,19^ followed by the glycopeptide, tetracycline, macrolide resistant. The results also support the high occurrence of the MDR efflux pump mechanism in the samples. Among the metal resistant genes, Cu, Ni, Co, As related were significantly varying between the sites revealing the probable presence of these metals in the sediments. Also, the most hits were observed against Cu, Hg, As, Ni, Fe with few genes related to Tungsten (W) and Tellurium (Te), similar trend was earlier reported in case of sediment samples, however, Ni and As were not among the dominant genes^13^.

As seen previously, the macrolide, tetracyclin and glycopepetide resistant genes were dominant in all the samples. The group of genes encoding resistance against glycopeptides *van*RA-RN were among the genes with >0.5% proportion, however, the proportion of macrolide resistant *car*A and *mac*B genes was highest in the samples (Fig. 4). Such results have also been reported previously in deep sediment samples^9^. Our observations were similar, wherein the abundance of *mac*B was co-related to that of *bcr*A gene, these respectively acting against the macrolides and peptide that interfere with important bacterial biosynthesis^20^.

The absence of more ARGs was observed in the GOK-TMS samples which may reflect the higher ARG diversity in the GOK-AFM samples owing to their high salinity (Supplementary Fig. 4). It has been recently reported that high salt stress condition increased the efflux of antibiotics in the *E. coli* by increasing the ARG expressions^21^. This could be one of the reasons for higher ARG diversity in the GOK-AFM samples and also the GOCS1 sample. Also, contaminants from anthropogenic activity tend to increase the ARGs and MDRs, which was also reflected in the GOCS1 and GOCS4 samples that have extensive activities as mentioned earlier^9^.

Total of 24 phyla observed in the sediment samples were further assessed for statistically significant variation between the three clusters observed in the Fig. 5b. As the samples were acting against specific groups of antibiotics, we assessed the corresponding microbial phyla variation in the same (p < 0.05) (Fig. 5a). The Cluster 1 which was supposedly more pristine had lower proportions of *Acidobacteria*. The most dominant taxa in sediments viz., Protobacteria was also the most abundant corresponding to encoding the ARGs^22^, however, its proportion was significantly lower in the Cluster 2 while the proportion of *Firmicutes* that is linked with human gut related ARGs was higher in Cluster 2^14^. This may suggest the mixing of human faeces in the samples and also the influence of human activities, as the cluster had huge number of significantly varying phyla. Further, similar analysis for genus level revealed significant features (those varying with p < 0.05 between the clusters) with highest abundance of *Thermotoga* (Fig. 5b). Most of the varying genus were extremophiles. The variations reflect the community of the concerned niche and thus their relevance to acquiring ARGs.

There were 22 biomarker ARGs (BM-ARGs) observed in the A and one in the GOC (Fig. 6a) i.e. *abc*A efflux pump acting against methicillin, daptomycin, cefotaxime, moenomycin drugs which are majorly antibiotics against the gram-positive microbes. This may reveal the in-flow of these in the GOC niche, however, these have not been abundantly reported from sediments in earlier reports. Also, the BM-ARGs in the A mostly belonged to the *gyr*A mutations conferring resistance to fluoroquinolones (Table 2). Open sea is hypothesized to be having different profile from the Gulfs, as the latter undergo constant sedimentation in varied way compared to the open sea. These BM-ARGs serve a preliminary point for assessing and monitoring the possible ARGs contaminants in the niche. Also, 24 biomarkers of bacterial community (BM-BC) were observed in the three regions (Fig. 6b). The GOK and A regions had BM-BC belonging to the *Proteobacteria* phyla with A having species belonging to alpha and gamma *Proteobacteria*, while GOK from gamma *Proteobacteria* along with one genus *Formosa*, from the *Bacteroidetes* phyla. *Acidobacteria*, *Proteobacteria* and *Bacteroidetes* have been commonly observed in ARG rich sediment samples owing to their high abundance^17^. The BM-BC observed in these are unique to marine and have fewer community members and thus may serve to be potential biomarkers. In GOK, *Enterobactericia* were among the predicted biomarkers which could owe to the hypothesis that wastewater source could be leading to carbepenem and β-lactam resistance in the pathogenic family, which has remained a challenge in the marine sediments as earlier observed^13,23^. In GOC, the observed BM-BC belonged to *Aquificae*, *Acidobacteria* and *Firmicutes*. The region stood unique as per earlier observations with different biomarkers and also those belonging to *Firmicutes*, which were abundant in the GOC samples, rather than any BM from the most dominant *Proteobacteria* phyla.

The present study, revealed the resistome of the pelagic sediments across the two Gulfs and compared it to an open sea coastline of Arabian sea of Kathiawar peninsula. The overall outcomes clearly showed the influence of both the niche environment and the on-shore activities on the ARG profile of the sediment microbiota. The most dominant ARGs included *car*A and *mac*B acting against macrolides, followed by 61 other ARGs with a proportion of >0.5% in at least one of the studied nine samples. These belonged to the ARGs against drug classes viz., glycopeptides, macrolides, and tetracycline. The nine samples seemed to form three major clusters with respect to ARGs and the proposed reason behind it seem to be the salinity variations and nearby on-shore anthropogenic activities. Efflux pump was the dominant mechanism of action in the observed ARGs. The corresponding bacterial community was dominated by *Proteobacteria* followed *Firmicutes* in one cluster, and *Actinobacteria* in the rest of the clusters. Significant variations in the clusters at both phyla and genus level revealed a core microbiome in the specific niche. Differentially abundant features among the samples from each region revealed 23 gene and 24 taxonomic biomarkers, which may be potentially validated further for their application in monitoring the specific/ similar niche for ARGs and antibiotic related contaminations.

## Methods

### Ethical statement

Due permissions from the Gujarat Maritime Board and Customs Division of Mandvi-Kutch and Morbi, Gujarat were obtained for the sampling of pelagic sediment cores from the Gulf of Kutch between 1-May-2017 to 20-May-2017 vide Sanction Letter Nos. M.K.B.U/PG/Project/18-19/17 dated 21.04.2017 and M.K.B.U/PG/Project/26-27/17 dated 25.04.2017. The sampling points (Supplementary Information 1) were selected such that no disturbance occurred to the Marine National Parks and any other Govt./ semi-Govt./ private projects in the region. The permissions for GOC and A samples were taken as described earlier^9^.

### Sampling sites

100 cm long core sediment samples below sea floor from 4 different coordinates (Supplementary Panel 1) of Gulf of Kutch [referred further as **GOKS1-4 **(**G**ulf **o**f **K**utch **S**ample **1** to **4**)] were collected by using a Small Free Fall Corer (#13.570, KC Denmark) and by sailing via hired fishing boats: ‘Ajmeri-I’ (Reg No.: IND-GJ-12M-81, Owner name: Sakinaben Amad Chamdiya, Mandvi-Kutch) ‘Nasib Apna Apna’ (Reg No.: IND-GJ-3-MO-141, Owner name: Amad Sidik Kakar, Navlakhi, Morbi) pre-equipped with mechanical winch. Detailed methodology is also described in Supplementary Panel 1. The sediment sampled from **T** (**T**op), **M** (**M**iddle) and **B** (**B**ottom) sections from the vertical core halves to the sterile containers and were stored at −80 °C until further use. Sample collection of **GOCS1-4** (**G**ulf **o**f Khambhat/ **C**ambay) and the open sea site **A** (**A**rabian sea) was as reported earlier^9^.

### Sediment physicochemical characteristics

The physicochemical parameters of the GOKS1-4 sediments (4 sites × 3 core sections = 12 samples i.e. GOKS1-4T, GOKS1-4M, GOKS1-4B) were estimated by mixing 10 g of initial sample in 50 mL of sterile MilliQ on an end to end tube shaker for 24 hours and measuring the parameters detailed in Supplementary Table 1 in triplicates using multiparameter (Model #PHS-MP30, BR Biochem, Life Sci.). The percent Total Organic Carbon (%TOC) was assessed in duplicates using Elementar (Germany) as per the manufacturer’s instructions.

### Metagenomic DNA extraction

Metagenomic DNA was extracted from each GOK sediments (4 sites × 3 core sections = 12 samples) using the PowerSoil DNeasy Kit (Qiagen, Germany) as described earlier^9^ and was quantified using Qubit Fluorometer v3.0 with Invitrogen™ Qubit™ dsDNA High Sensitivity assay kit (ThermoFicher Scientific).

### High-throughput sequencing of metagenomic DNA

The high-quality DNA from the three corer sections viz., T, M and B from each sampling point of GOK were pooled in equal concentrations and processed for metagenomic shotgun sequencing by the Trueseq Nano kit as per the manufacturer’s protocol. The library was assessed for quality and mean base-pair length followed by sequencing on the HiSeq. 4000 (Macrogen Inc., Seoul, Rep. of Korea).

### Metagenomic data analysis

The generated paired-end raw reads were quality checked by FastQC^24^ and filtered for adaptor sequences, short read removal by the Scythe (v0.994) and Sickle^25,26^. The filtered sequences (almost 96.5% of the total raw reads on an average were retained) from each sample were processed for De Novo assembly by the CLC Genomics Workbench v.11 (Qiagen, USA), all the parameters except a high k-mer value of 31 were kept default. The obtained scaffolds were then subjected to ORF prediction using MetaProdigal^27^. The predicted ORFs in each of the four GOK samples were individually blasted (e-value threshold of 1e-5) against the protein sequences reference file, that comprised of 2,893unique ARG sequences downloaded from the Comprehensive Antibiotic Resistance Database (CARD, https://card.mcmaster.ca/)^28^. The samples were normalized by computing the percentage of ARG hits to understand the complexity of the sample based on the number of unique ARGs under each sample. Similarly, the samples from the studied sites were assessed for metal resistance against the BacMet database^29^. The contigs showing hits for ARGs were extracted and checked for the marine microbial taxa by performing blastn against the MarRefDB^30^. The five samples from GOC and A sites were also processed by same pipeline^9^ and their raw results were considered for comparison between the three regions for antibiotic resistance mechanisms, drug classes against which the ARGs were observed, ARG diversity, ARG enriched microbiota profiling at phylum and genus levels and biomarkers prediction.

### Statistical analysis

Significant variation (p < 0.05) in the ARG abundance as well as the corresponding microbiota analysis was assessed with following variables: sites, samples near the creek (referred as **AFM: A**way **F**rom **M**outh) and those near the mouth (referred as **TMS: T**owards **M**outh **S**ide) of Gulfs, using two-way Welch’s t-test with a Benjamin-Hochberg FDR correction using STAMP (Statistical Analysis of Metagenomic Profiles) v1.07 software package^31^. To identify niche-specific biomarkers by LEfSe analysis, the Kruskal-Wallis test (alpha value of 0.05 for ARG; alpha value of 0.1 and 0.05 for microbial taxonomy) was performed with LDA score of >2 at multiple taxonomic levels using the linear discriminant analysis (LDA) effect size (LEfSe)^5^. For input, the microbiota abundance profiles were computed at each taxonomic levels from phylum to species and the ARG abundance profile was assessed based on only the ARO (Antibiotic Resistance Ontology) category hits.

## Supplementary information

Supplementary Figure S1-S6, Table S1-S2

## Data Availability

The metagenomic sequence of the four GOK samples in the study has been deposited to the EBI metagenomics under the BioProject PRJEB26615.
