# Supplementary material for: Antibiotic Resistome Biomarkers associated to the Pelagic Sediments of the Gulfs of Kathiawar Peninsula and Arabian Sea
Source: Sci Rep. 2019 Nov 21;9:17281. doi: 10.1038/s41598-019-53832-9 (PMC6872816; doi:10.1038/s41598-019-53832-9)
Supplement: Supplementary file 1 — Supplementary Figure S1-S6, Table S1-S2 [file 41598_2019_53832_MOESM1_ESM.pdf]

Antibiotic Resistome Biomarkers associated to the Pelagic Sediments of the Gulfs of Kathiawar Peninsula and Arabian Sea

Chandrashekar Mootapally[1,5#*], Neelam M. Nathani[2,5#], Paresh Poriya[3], Imtiyaz Beleem[2], Jignesh C. Dabhi[1],Indra R. Gadhvi[1], Chaitanya G. Joshi[4,5]

[1] Department of Marine Science, Maharaja Krishnakumarsinhji Bhavnagar, University, Bhavnagar - 364-001, Gujarat, India

[2] Department of Life Sciences, Maharaja Krishnakumarsinhji Bhavnagar University, Bhavnagar - 364-001, Gujarat, India

[3] Government Science College, Bhakta Kavi Narsinh Mehta University,Veraval- 362-265, Gujarat, India

[4] Department of Animal Biotechnology, College of Veterinary & Animal Husbandry, Anand Agricultural University, Anand - 388-001, Gujarat, India

[5] Gujarat Biotechnology Research Centre (GBRC), Department of Science & Technology Government of Gujarat,Gandhinagar - 382-011,Gujarat, India

# Authors have contributed equally.

*Corresponding Authors:

Dr. Chandrashekar Mootapally,
Department of Marine Science,
Maharaja Krishnakumarsinhji Bhavnagar,
University, Bhavnagar - 364-001, Gujarat, India
E-mail: chandu.avi@gmail.com

Supplementary Information 1: (a) Sampling regions of study with the 4 Gulf of Kutch sites indicated (Mapdata: Google, Imagery; Attribution: Map data©2017 Google Imagery ©2017 TerraMetrics). (b) Sampling site details (Wherein GOKS1-4 stands for Gulf of Kutch Sample 1 to Sample 4) (c) Sampling methodology

(a)

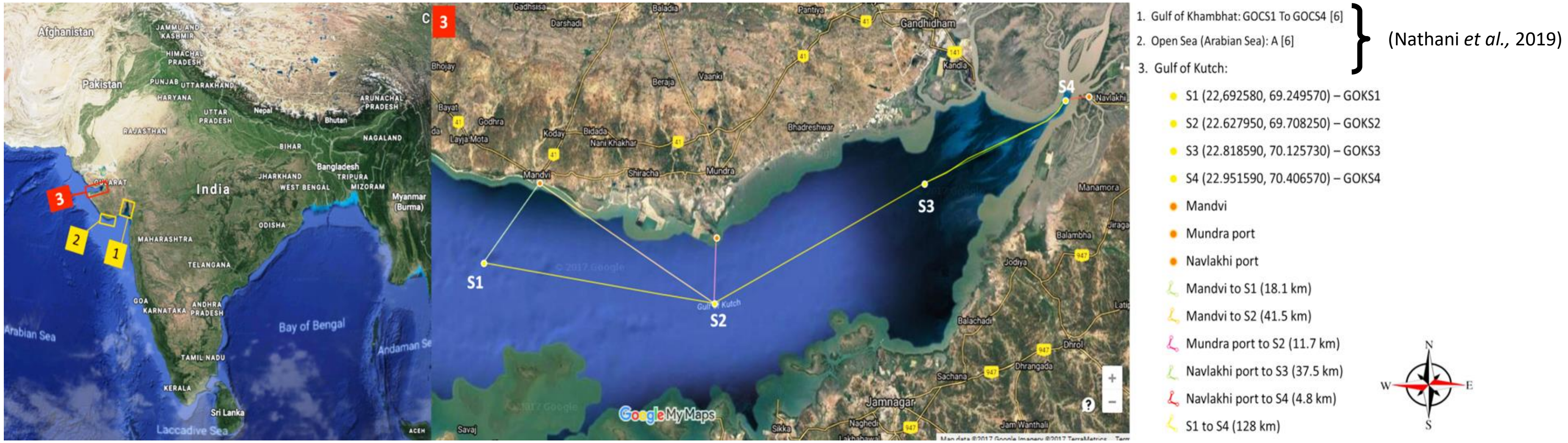

(b)

Sample	Collection Date	Time	Latitude	Longitude	Depth	Elevation
GOKS1	03.05.2017	11:15 am	22°41'33.3"N	69°14'58.5"E	32m	-48m
GOKS2	04.05.2017	12:06 am	22°37'40.6"N	69°42'29.7"E	28m	-40m
GOKS3	08.05.2017	04:10 pm	22°49'06.9"N	70°07'32.6"E	15m	-10m
GOKS4	09.05.2017	05:05 pm	22°57'05.7"N	70°24'23.6"E	5m	-5m

(c)

After the 100 cm corer sediments were collected, the internal pp liner was dismantled from the corer sampler and the pp liner edges were sealed. These were further stored in customized (>1m in length) insulated ice cabinet to maintain the sediment samples under low temperature during the sail and immediately transferred to deep freezer upon reaching the laboratory until further processing. The internal pp liners of each sediment samples were cut down vertically into two equal sections with utmost care taken to not disturb the sediments and then the sediment was separated into two halves vertically using thin metal wire by slipping right down through the vertical core length with the purpose to get a clear view of sediment texture and color for proper documentation. The sediment were further transferred into sterile containers by collecting 10 cm portions each from Top (T), Middle (M) and Bottom (B) sediments in the vertical core halves as shown below with care taken that sediment from different lengths do not mix.

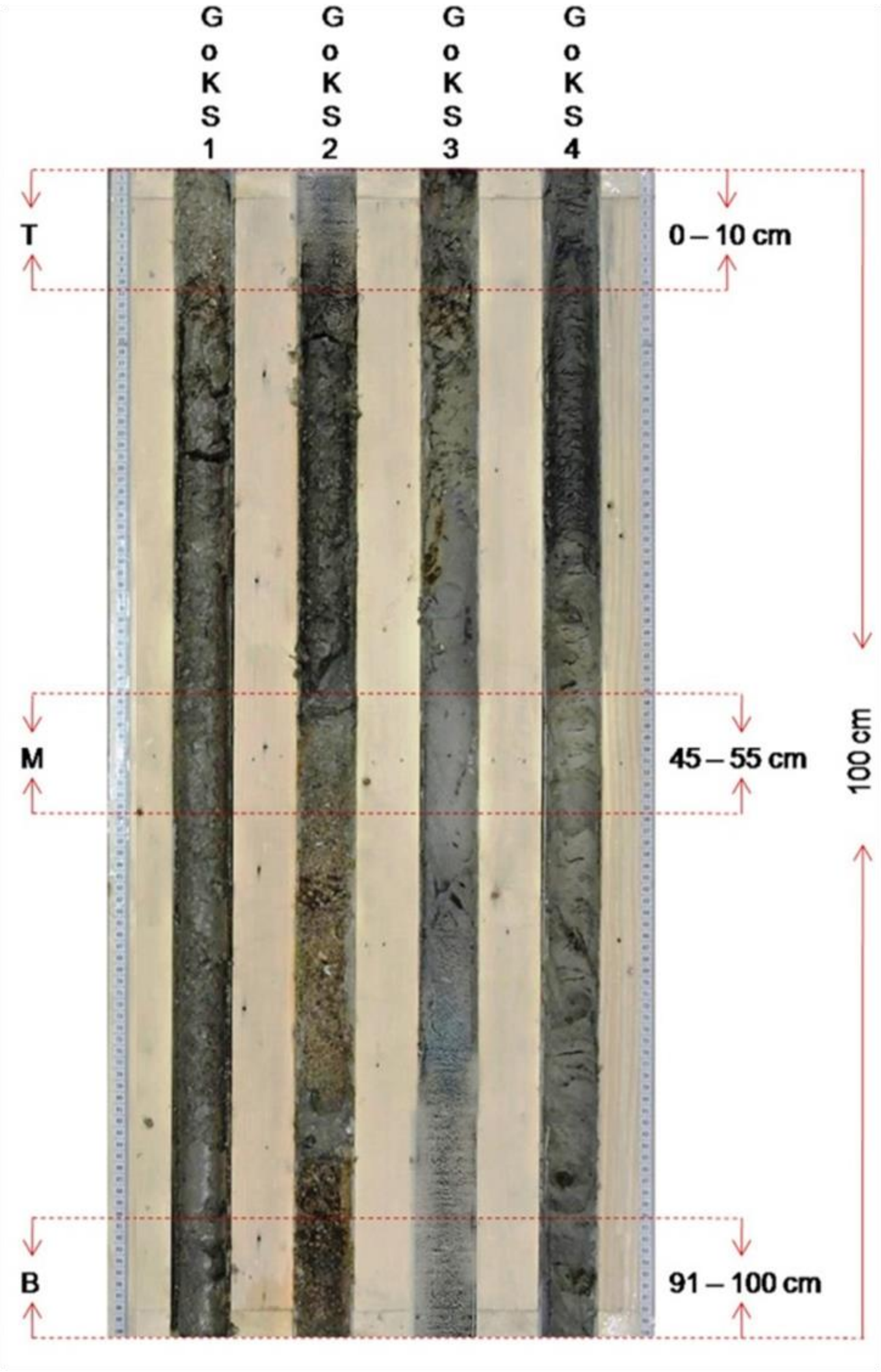
GoKS1
GoKS2
GoKS3
GoKS4
T
M
B
0 – 10 cm
45 – 55 cm
91 – 100 cm
100 cm

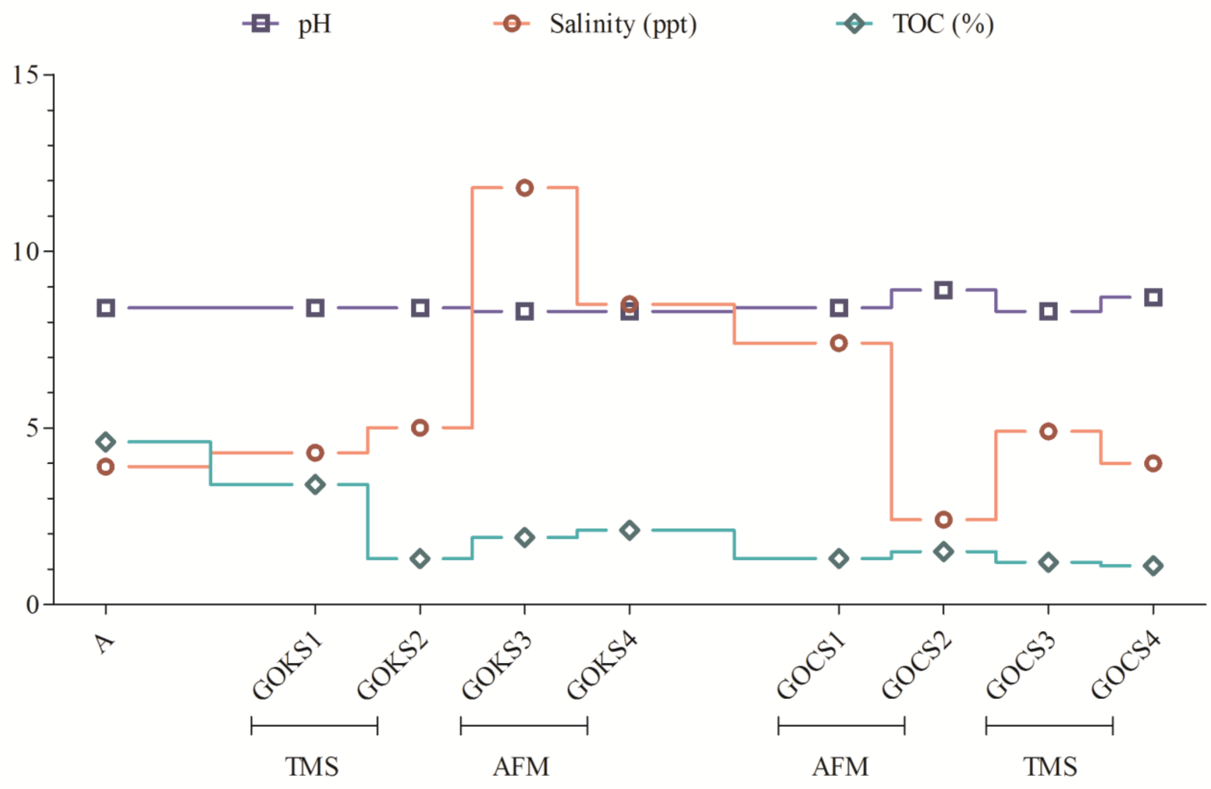

Supplementary Figure 1: Trend of the major physico-chemical parameters in the studied samples (values are mean of top, middle and bottom of respective samples)

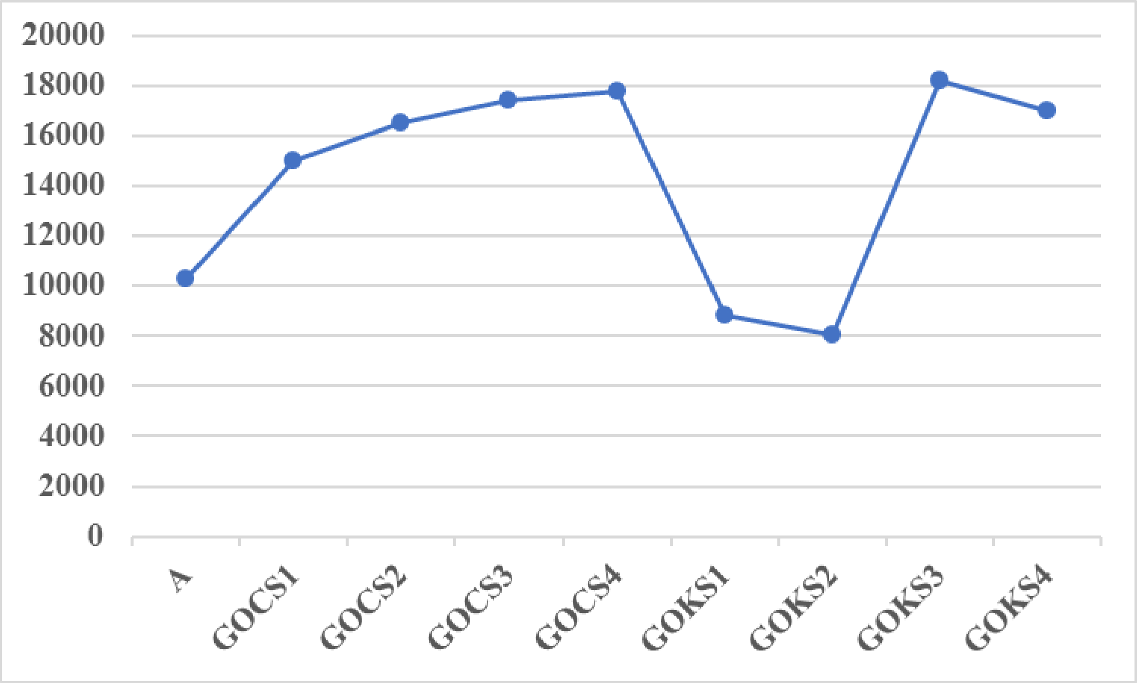

Supplementary Figure 2: Hits to bacmet genes across the studied samples

Supplementary Figure 3. Significantly varying metal resistant genes between (a) Cluster 1 and other samples (b) Cluster 2 and other samples (c) Cluster 3 and other samples

(a)

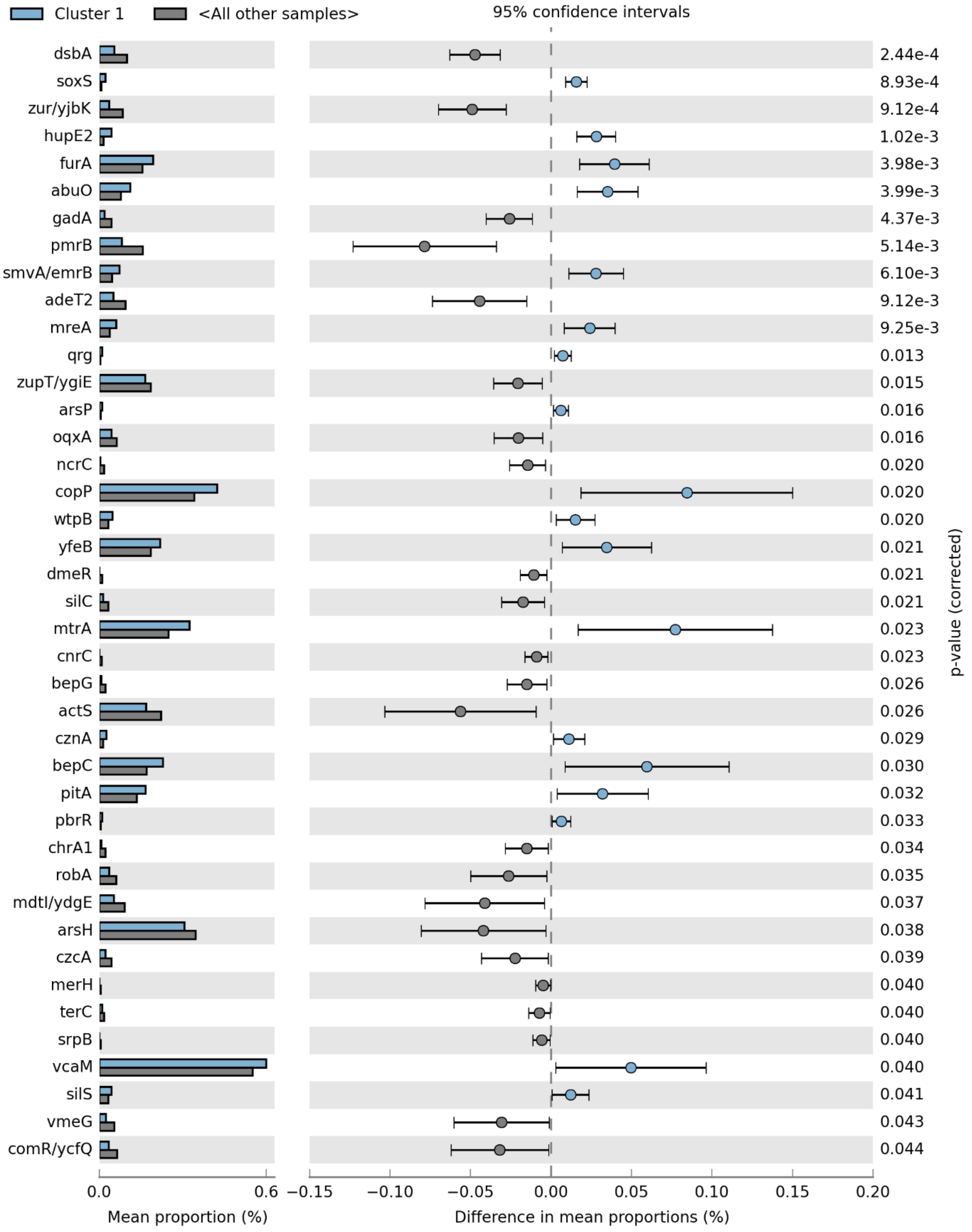

(b)

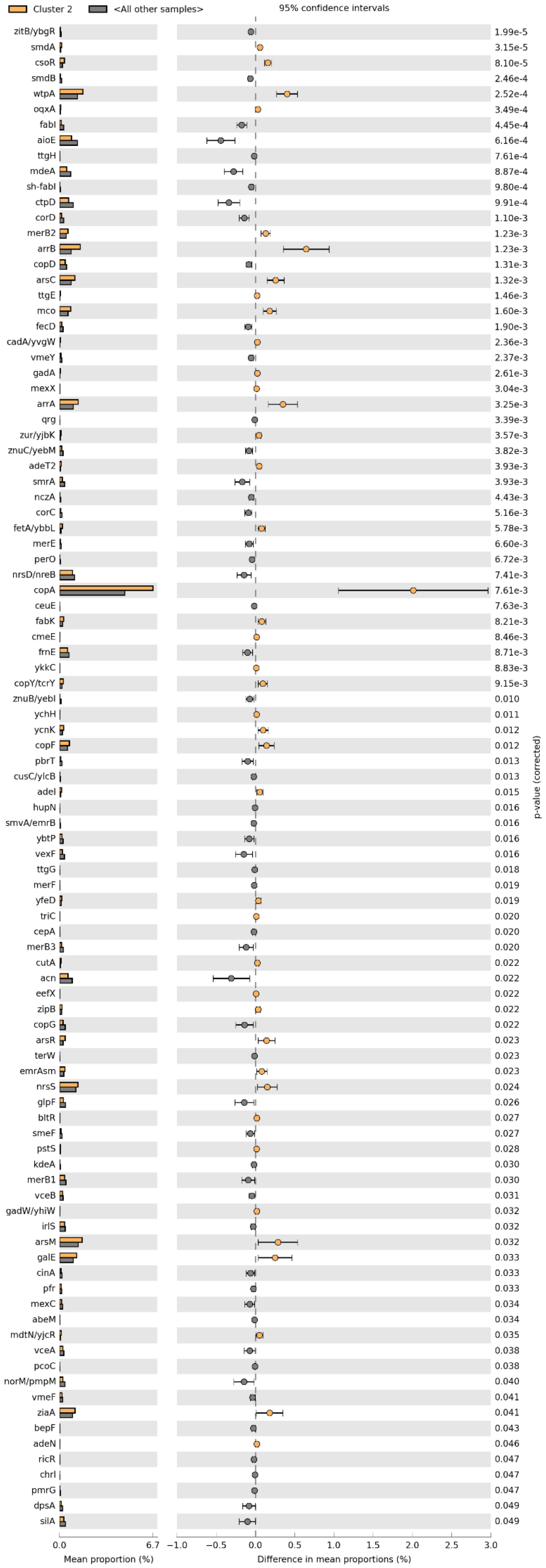

(C)

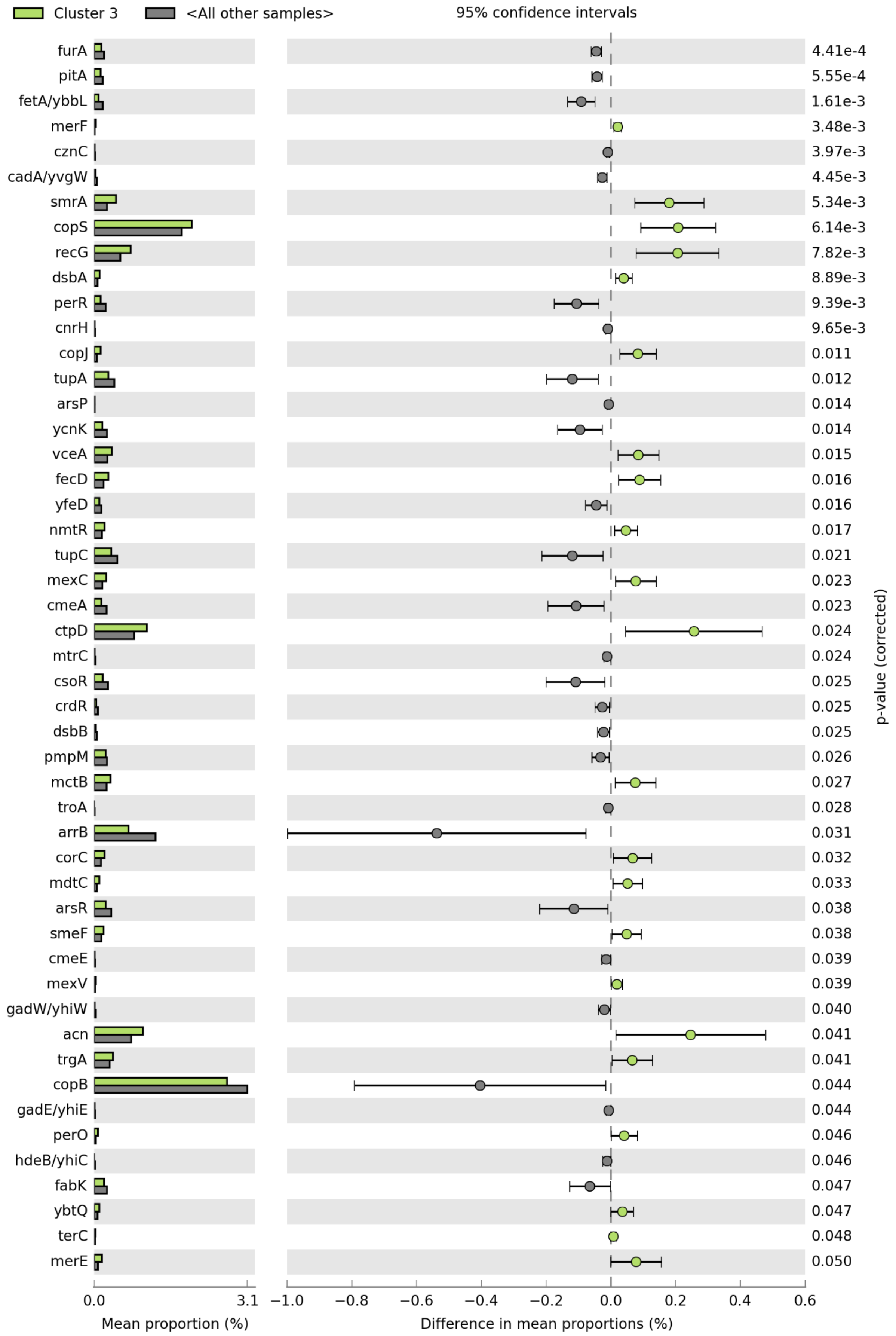
Cluster 3
<All other samples>
95% confidence intervals
furA 4.41e-4
pitA 5.55e-4
fetA/ybbL 1.61e-3
merF 3.48e-3
cznC 3.97e-3
cadA/yvgW 4.45e-3
smrA 5.34e-3
copS 6.14e-3
recG 7.82e-3
dsbA 8.89e-3
perR 9.39e-3
cnrH 9.65e-3
copJ 0.011
tupA 0.012
arsP 0.014
ycnK 0.014
vceA 0.015
fecD 0.016
yfeD 0.016
nmtR 0.017
tupC 0.021
mexC 0.023
cmeA 0.023
ctpD 0.024
mtrC 0.024
csoR 0.025
crdR 0.025
dsbB 0.025
pmpM 0.026
mctB 0.027
troA 0.028
arrB 0.031
corC 0.032
mdtC 0.033
arsR 0.038
smeF 0.038
cmeE 0.039
mexV 0.039
gadW/yhiW 0.040
acn 0.041
trgA 0.041
copB 0.044
gadE/yhiE 0.044
perO 0.046
hdeB/yhiC 0.046
fabK 0.047
ybtQ 0.047
terC 0.048
merE 0.050
0.0
3.1
Mean proportion (%)
−1.0 −0.8 −0.6 −0.4 −0.2 0.0 0.2 0.4 0.6
Difference in mean proportions (%)
p-value (corrected)

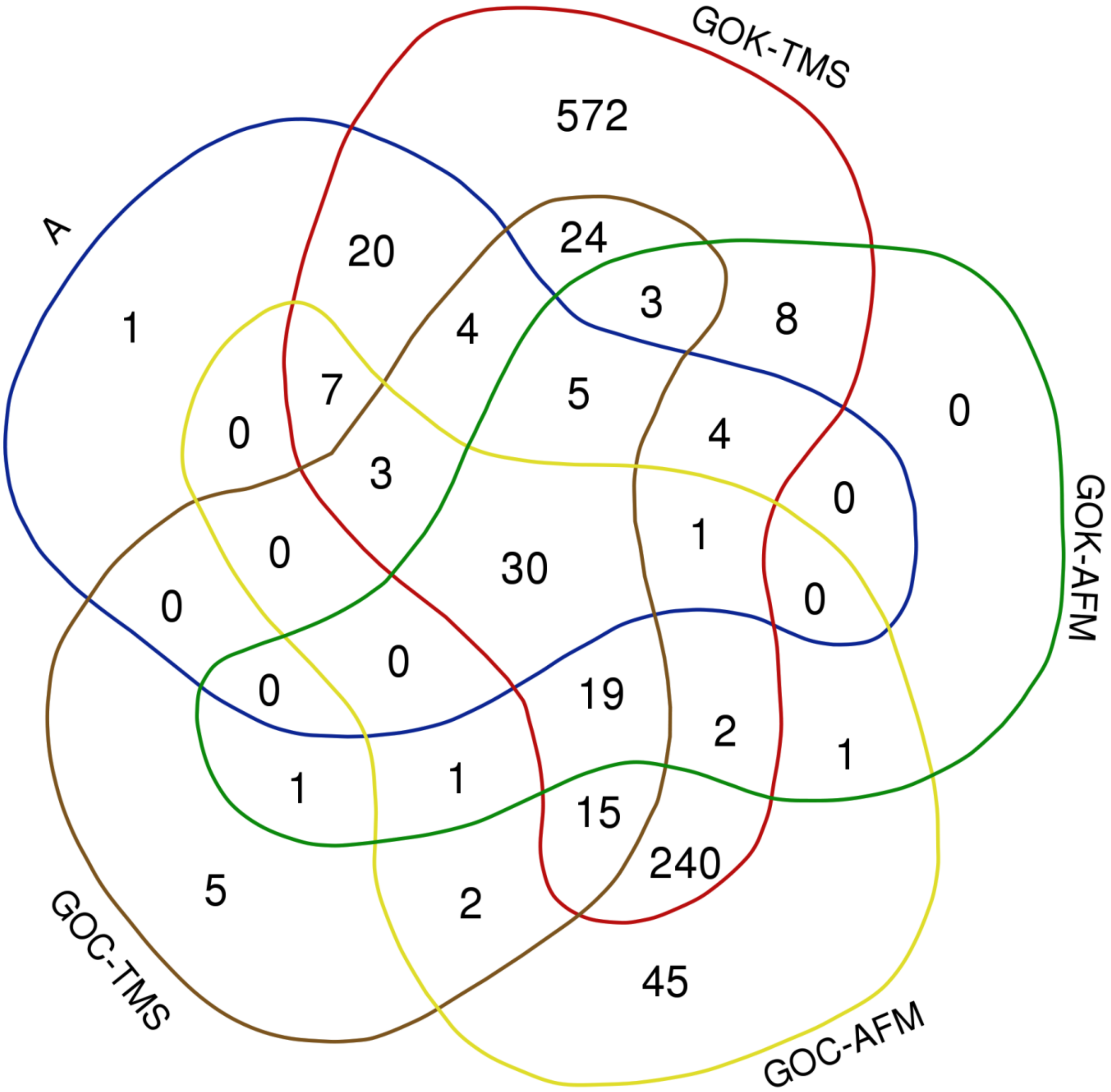

Supplementary Figure 4: Venn diagram showing the unique and shared ARGs absent among studied samples (the comparison was considered between **A** and following groups **GOK-TMS**: GOKS1 and S2; **GOK-AFM**: GOKS3 and S4; **GOC-AFM**: GOCS1 and S2; **GOC-TMS**: GOCS3 and S4)

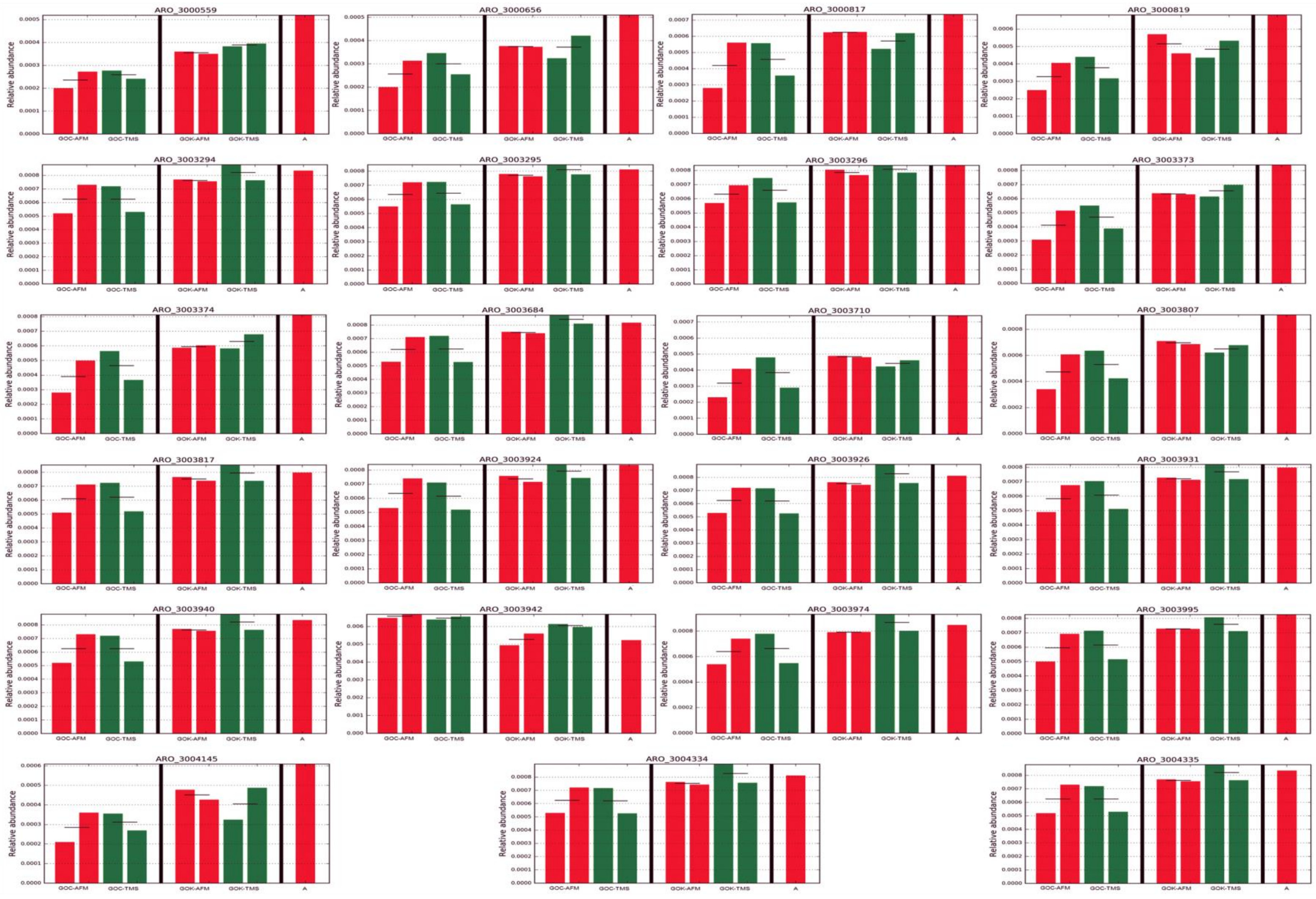

Supplementary Figure 5: Proportion of individual differentially expressed biomarker ARGs in subclasses (GOK-TMS, GOK-AFM, GOC-TMS and GOC-AFM)

Supplementary Table 1: Physicochemical properties of the studied sediment samples (mean±SD)

Sample ID	pH	EC (mS)	TDS (ppt)	Salinity (ppt)	Res (Ohm)	%TOC
AT	8.47±0.02	7.45±0.05	3.73±0.1	4.10±0.05	133±3	4.74±0.02
AM	8.53±0.01	5.04±0.07	2.52±0.00	2.70±0.02	197±1	3.27±0.02
AB	8.05±0.01	8.58±0.10	4.28±0.00	4.77±0.03	116±10	5.65±0.03
GOKS1T	8.48±0.01	7.26±0.02	3.63±0.00	4.01±0.01	137±4	6.49±0.03
GOKS1M	8.47±0.02	7.27±0.01	3.64±0.00	4.00±0.03	136±3	1.80±0.00
GOKS1B	8.35±0.01	8.53±0.03	4.28±0.01	4.76±0.03	116±2	1.86±0.01
GOKS2T	8.41±0.02	12.30±0.05	6.15±0.01	7.03±0.04	81±1	1.45±0.00
GOKS2M	8.12±0.01	10.05±0.03	5.02±0.01	5.69±0.01	98±1	1.84±0.01
GOKS2B	8.57±0.01	4.39±0.00	2.22±0.00	2.38±0.00	222±3	0.55±0.00
GOKS3T	8.09±0.01	20.80±0.04	10.43±0.03	12.51±0.02	47.8±1	1.87±0.01
GOKS3M	8.51±0.02	20.00±0.03	9.99±0.02	11.93±0.02	49.9±2	1.63±0.01
GOKS3B	8.33±0.01	18.65±0.03	9.31±0.02	11.01±0.01	53±8	2.21±0.02
GOKS4T	8.09±0.00	11.65±0.01	5.82±0.00	6.63±0.01	85±2	2.14±0.03
GOKS4M	8.35±0.01	12.60±0.01	6.30±0.01	7.21±0.02	79±1	2.27±0.02
GOKS4B	8.49±0.01	19.58±0.03	9.80±0.02	11.65±0.02	51±1	1.90±0.01
GOCS1T	8.32±0.00	13.40±0.02	6.71±0.03	7.73±0.05	74±5	1.30±0.00
GOCS1M	8.30±0.01	13.59±0.05	6.79±0.01	7.86±0.04	73±9	1.37±0.00
GOCS1B	8.44±0.03	11.49±0.05	5.75±0.02	6.54±0.05	87±3	1.29±0.00
GOCS2T	8.99±0.01	3.69±0.06	1.86±0.00	1.95±0.03	270±2	1.64±0.00
GOCS2M	8.88±0.00	4.37±0.03	2.19±0.01	2.33±0.01	227±3	1.28±0.00
GOCS2B	8.73±0.01	5.34±0.02	2.68±0.04	2.90±0.01	185±3	1.70±0.02
GOCS3T	8.10±0.02	11.02±0.03	5.52±0.01	6.26±0.03	90±3	1.14±0.01
GOCS3M	8.48±0.01	6.82±0.08	3.41±0.00	3.73±0.02	146±7	1.24±0.01
GOCS3B	8.34±0.02	8.48±0.05	4.24±0.02	4.67±0.02	118±8	1.24±0.01
GOCS4T	8.85±0.01	5.46±0.03	2.73±0.01	2.94±0.01	182±6	1.07±0.01
GOCS4M	8.45±0.01	9.26±0.01	4.65±0.00	5.21±0.03	107±5	0.96±0.00
GOCS4B	8.68±0.02	6.77±0.06	3.40±0.02	3.73±0.01	146±4	1.21±0.01

EC: Electrical Conductivity; TDS: Total Dissolved Salts; Res: Resistance; TOC: Total Organic Carbon; mS: Milli Simon; ppt: parts per trillion.

Supplementary Table 2: Abundance (%) of observed phyla in the samples

Phylum	A	GOKS1	GOKS2	GOKS3	GOKS4	GOCS1	GOCS2	GOCS3	GOCS4
Acidobacteria	0.777654	0.817228	0.928162	0.761682	1.115457	1.282692	0.937192	1.514857	1.156734
Actinobacteria	13.58752	9.130458	5.2438	4.854996	6.900127	4.242318	12.43604	7.989602	4.716442
Aquificae	0.479655	0.509582	0.778204	0.522001	0.494827	1.036995	0.597059	0.624468	0.932349
Bacteroidetes	7.085634	6.394167	6.274566	11.5609	6.736202	8.73878	6.355243	6.676427	8.431541
Calditrichaeota	0.966455	0.830781	1.72373	1.615102	0.980504	2.099335	1.211635	1.168263	1.926233
Candidatus	0.011226	0.014908	0.026835	0.014213	0.007624	0.032384	0.005839	0.008964	0.040402
Chlorobi	0.865421	0.818583	1.322789	1.26624	0.852414	1.316484	0.886829	0.955376	1.23443
Crenarchaeota	0.200027	1.410837	1.659011	0.423157	0.24627	1.787462	0.597788	0.277873	1.954203
Cyanobacteria	4.89963	4.041417	6.823886	4.717389	4.822465	5.406737	5.05748	4.909841	5.25161
Deferribacteres	0.725606	0.618003	1.07812	0.783001	0.844027	1.339012	0.764206	0.817186	1.302802
Deinococcus-Thermus	0.71438	0.544819	0.798725	0.534276	0.761683	0.834947	0.892668	0.599818	0.848437
Euryarchaeota	4.716952	8.909549	16.67535	6.711717	4.622704	16.67852	9.250027	5.0555	17.27145
Firmicutes	8.657271	7.218171	12.82379	6.712363	7.735005	13.8604	8.606255	7.132826	13.27292
Fusobacteria	0.137773	0.191093	0.252561	0.144713	0.189849	0.290753	0.14306	0.181514	0.271003
Nanoarchaeota	0	0.001355	0.006314	0.001938	0.000762	0.006336	0.00073	0.001494	0.006216
Planctomycetes	0.097972	0.117909	0.232041	0.105305	0.131141	0.324545	0.105106	0.22185	0.26603
Proteobacteria	52.81517	51.8635	36.31829	54.16276	59.43716	32.06941	46.71289	56.16998	32.9722
Spirochaetes	0.392909	0.429621	0.722956	0.573684	0.41477	0.703298	0.456188	0.408593	0.668805
Synergistetes	0.174513	0.224975	0.288867	0.169263	0.154014	0.396353	0.231378	0.220356	0.397181
Tenericutes	0.035719	0.042013	0.096289	0.03424	0.04041	0.044352	0.033575	0.04183	0.045996
Thaumarchaeota	0.191862	2.234841	1.002352	1.588614	0.991179	1.652997	1.694099	2.454547	1.593073
Thermodesulfobacteria	0.43271	0.363213	0.778204	0.443184	0.429257	0.960963	0.498522	0.519145	0.918053
Thermotogae	1.684917	2.232131	3.591104	1.881917	1.577499	4.278222	2.20138	1.58582	4.030233
Verrucomicrobia	0.349026	1.040848	0.554056	0.417342	0.51465	0.616706	0.324806	0.463869	0.491659
